# Metagenomic next-generation sequencing facilitates precision treatment and prognostic improvement in pulmonary cryptococcosis

**DOI:** 10.3389/fcimb.2025.1638215

**Published:** 2025-10-23

**Authors:** Yapeng Xu, Jingxuan Miao, Jing Chen, Liqun Ye, Kangli Yang, Hongmin Wang

**Affiliations:** Department of Respiratory Medicine, The First Affiliated Hospital of Zhengzhou University, Zhengzhou, China

**Keywords:** pulmonary cryptococcosis, metagenomic next-generation sequencing, diagnosis, treatment, prognosis

## Abstract

**Background:**

The early diagnosis of pulmonary cryptococcosis (PC) remains challenging due to the low sensitivity and prolonged turnaround time of conventional diagnostic methods. Despite the broad-spectrum pathogen detection capability of metagenomic next-generation sequencing (mNGS), its clinical utility in the diagnosis and therapeutic management of pulmonary cryptococcosis remains underexplored.

**Methods:**

In this retrospective study, 31 patients diagnosed with *Cryptococcus* infection through mNGS at The First Affiliated Hospital of Zhengzhou University between July 2023 to March 2025 were included. data on clinical characteristics, treatment regimens, and patient prognosis were systematically collected.

**Results:**

Compared to conventional pathogen detection methods, mNGS demonstrated superior sensitivity, shorter turnaround time (1.00 d vs. 4.50 d, *p* = 0.002), and significantly reduced interval from admission to clinical decision-making (3.50 d vs. 9.00 d, *p* = 0.002). Among 31 patients with mNGS-identified cryptococcal infection, only 12 underwent fungal culture, with merely 1 case yielding positive results (positivity rate: 8.33%). Antimicrobial therapy was optimized for all patients based on mNGS findings. During post-discharge follow-up of 27 cases, 1 patient experienced disease recurrence, 1 died from tumor metastasis, and 1 was lost to follow-up.

**Conclusion:**

Our retrospective analysis revealed that mNGS facilitated treatment optimization, improved clinical outcomes, and provided crucial evidence supporting the precision management of pulmonary cryptococcosis.

## 1 Introduction

As environmental fungi commonly found in nature, *Cryptococcus gattii* or *Cryptococcus neoformans* are the primary causes of pulmonary cryptococcosis, a fungal infection (Setianingrum et al., 2019; Howard-Jones et al., 2022). Transmission typically occurs through the respiratory tract. Once inhaled, *Cryptococcus* can proliferate in the lungs, leading to pulmonary infection (Li et al., 2019). *Cryptococcus neoformans* is ubiquitously distributed worldwide, primarily transmitted through natural environmental reservoirs such as air and soil. droppings. It largely infects individuals with compromised immune function, including those with HIV/AIDS, organ transplants, or undergoing immunosuppressive therapy (Soltani et al., 2013; Diniz-Lima et al., 2022). In recent years, the incidence of pulmonary cryptococcosis in immunocompetent individuals has gradually increased (Hou et al., 2019). Pulmonary cryptococcosis is emerging as a significant infectious disease with serious implications for public health (Zhao et al., 2023).

Early diagnosis of pulmonary cryptococcosis is of significant clinical importance, but it is often prone to misdiagnosis as lung cancer, pulmonary tuberculosis, or other fungal pneumonias due to the nonspecific clinical manifestations and limitations of traditional diagnostic methods (Ekeng et al., 2022). Without timely and targeted intervention, the disease can progress rapidly (Jarvis and Harrison, 2008), leading to irreversible lung damage and potentially disseminating to the central nervous system (CNS) and other organs (Salazar et al., 2020). Delays in initiating antifungal treatment not only worsen clinical outcomes (Bratton et al., 2013) but also risk the development of drug-resistant cryptococcal strains due to prolonged exposure to subtherapeutic drug levels (Arastehfar et al., 2020). Therefore, timely and accurate diagnosis of pulmonary cryptococcosis is necessary for initiating appropriate treatment, optimizing therapeutic strategies, and improving patient outcomes (McCarthy and Walsh, 2017; Arastehfar et al., 2020).

mNGS offers several advantages over conventional diagnostic approaches, including its independence from culture, rapid identification of low-abundance pathogens, and high sensitivity in identifying co-infections (Qian et al., 2020). As an unbiased microbial diagnostic method (Kan et al., 2024), mNGS has demonstrated potential in the early diagnosis of infectious diseases. However, its sensitivity and diagnostic reliability remain to be fully validated through further research and clinical comparison with standard diagnostic techniques (Liu et al., 2025).

**Figure 1:**
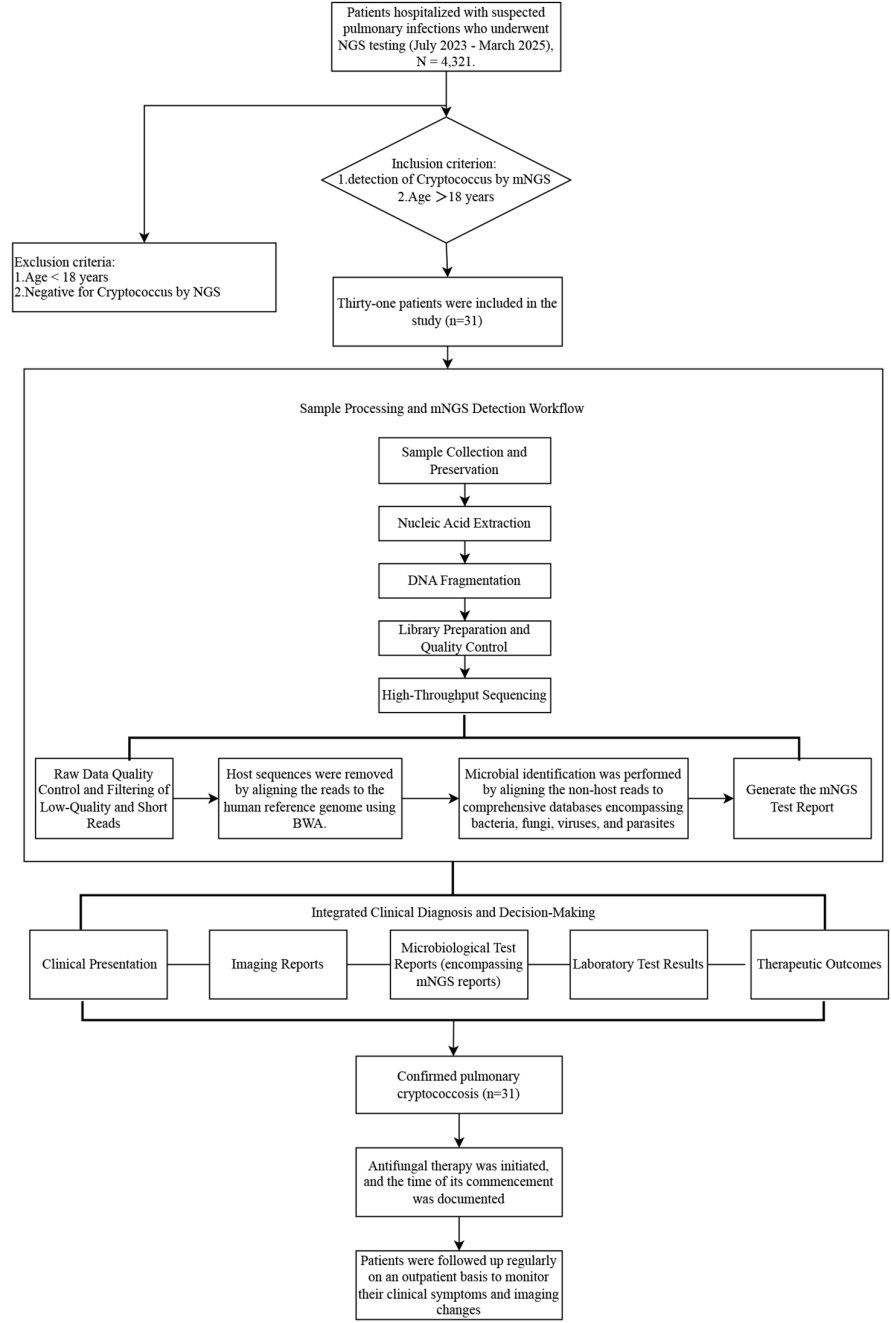
Study design and workflow.

In this retrospective study, we compared mNGS with traditional pathogen detection methods and recorded changes in antimicrobial agents before and after patient examination to evaluate its value in guiding treatment decisions. Although *cryptococcus* pathogens can be promptly detected and taxonomically characterized via mNGS, providing critical data for guiding therapeutic strategies, validation of its impact on patient prognosis based on patient outcomes is still needed (Huang et al., 2024). The objective of this study is to evaluate the diagnostic sensitivity and clinical utility of mNGS in managing pulmonary cryptococcosis by analyzing integrated patient medical records (symptomatology, past medical history, and radiologic data) and mNGS-derived microbial profiles from 31 patients diagnosed with pulmonary cryptococcosis between July 2023 to March 2025. To clearly present the research design framework, the overall study flowchart is shown in **Figure 1**.

## 2 Materials and methods

### 2.1 Patients

This retrospective study included 31 patients who tested positive for *Cryptococcus* via mNGS at The First Affiliated Hospital of Zhengzhou University between July 2023 to March 2025. The inclusion criteria for this study were as follows: hospitalized patients with suspected pulmonary infection at the First Affiliated Hospital of Zhengzhou University between July 2023 and March 2025, with *Cryptococcus* infection confirmed by mNGS testing. Exclusion criteria included: (1) age under 18 years; (2) absence of *Cryptococcus* detection on mNGS or missing clinical data.

In this study, the diagnosis of pulmonary cryptococcosis was established based on a comprehensive assessment incorporating the 2024 edition of the Global Guideline for the Diagnosis and Management of Cryptococcosis: An Initiative of the ECMM and ISHAM in Cooperation with the ASM and the clinical profiles of individual patients. Based on routine clinical diagnostic practices, we established a composite clinical reference standard to define confirmed cryptococcal pneumonia. A patient is considered a true positive if they meet any of the following criteria: A. Microbiological confirmation: Bronchoalveolar lavage fluid (BALF) or tissue fungal culture positive for Cryptococcus; B. Pathological confirmation: Histopathological examination of lung tissue revealing Cryptococcus; C. mNGS-based confirmation: Detection of Cryptococcus by mNGS in BALF or tissue samples, with clinical interpretation supported by read count, relative abundance, and other relevant metrics; D. Clinical–imaging confirmation: Presence of relevant clinical symptoms (e.g., fever, cough), pulmonary imaging highly suggestive of invasive fungal infection (e.g., nodules, halo sign, cavities), exclusion of other major pathogens (e.g., Mycobacterium tuberculosis, common bacteria), and significant clinical improvement following anti-cryptococcal therapy.

The study was conducted in accordance with the ethical principles of the Declaration of Helsinki and received approval from the Institutional Review Board of the First Affiliated Hospital of Zhengzhou University (Approval No. 2024-KY-0615-001). Given the retrospective nature of the research and the use of anonymized data analysis protocols, the requirement for informed consent was waived by the ethics committee.

### 2.2 Therapeutic regimen

Patients enrolled in this study had localized pulmonary cryptococcal infection without involvement of the central nervous system or other organs, consistent with a diagnosis of isolated pulmonary cryptococcosis. Treatment strategies were stratified based on immune status and disease severity: Mild pulmonary cryptococcosis: Oral fluconazole 400–800 mg/day for 6–12 months. Severe pulmonary cryptococcosis: Induction phase: Liposomal amphotericin B (3–4 mg/kg/day) combined with flucytosine (25 mg/kg/day) for 2 weeks; alternatively, a single dose of liposomal amphotericin B (10 mg/kg) combined with flucytosine (1200 mg/day) may be used. Consolidation phase: Fluconazole 400–800 mg/day for 8 weeks. Maintenance phase: Fluconazole 200 mg/day for 12 months. In cases of fluconazole resistance or intolerance, voriconazole or posaconazole was administered as an alternative. The diagnosis of cryptococcal infection was comprehensively determined based on mNGS results, clinical manifestations, laboratory tests, microbiological studies, and imaging findings. All diagnosed patients received antifungal therapy according to the above protocol (Chang et al., 2024).

### 2.3 Diagnostic process efficiency

To evaluate clinical workflow efficiency, we analyzed two key time intervals. The turnaround time (TAT) was defined as the period from specimen receipt in the laboratory (T1) to the delivery of the final report to the clinician (T4). To ensure a fair and accurate comparison between fungal culture and mNGS methods, the following standardized time points were established:T1 – Specimen Receipt Time: Time of sample registration in the laboratory.T2 – Processing Start Time: Fungal culture: inoculation onto culture media; mNGS: initiation of nucleic acid extraction or sequencing.T3 – Analysis-Ready Time: Fungal culture: appearance of visible colonies allowing identification; mNGS: completion of sequencing and generation of FASTQ files.T4 – Report Issuance Time: Time when the clinical physician receives the final report. Thus, TAT was uniformly calculated as the duration from T1 to T4 for both methods. Additionally, the result time was evaluated as the interval from patient admission to the receipt of mNGS or fungal culture results. This metric reflects the time from admission to clinical decision-making based on diagnostic outcomes (Wang and Xing, 2023).

### 2.4 Sample collection and mNGS workflow

#### 2.4.1 Sample collection

Three types of clinical specimens were collected, comprising bronchoalveolar lavage fluid (BALF, n=26), lung tissue (n=4), lung puncture fluid (n=1). BALF and lung puncture fluid samples exceeding 5 mL were sent to the laboratory within 2 hours of collection at room temperature. If a delay was anticipated, samples were refrigerated at 2-8 °C. In addition to mNGS, routine microbiological tests were also performed for BALF specimens, based on patients’ clinical history. These included fungal cultures, Gomori’s methenamine silver (GMS) staining, and immunofluorescence staining. Lung tissue samples larger than 3x3x3 mm³ were preserved in cryovials with 5 mL dry ice and transported under frozen conditions.

#### 2.4.2 DNA extraction and library preparation

##### 2.4.2.1 DNA extraction

Total DNA was extracted using the Nucleic Acid Extraction Kit (Cat. No. MD013; MatriDx Biotech Corp., Hangzhou, China). The extraction procedure was performed on the NGS Automatic Library Preparation System (Cat. No. MAR002; MatriDx Biotech Corp.) to ensure automated and standardized processing.

##### 2.4.2.2 Library preparation

Different library preparation kits were selected based on sample type:Blood samples: Cell-free DNA Library Preparation Kit (Cat. No. MD007); Other samples (tissue, body fluids, etc.): Total DNA Library Preparation Kit (Cat. No. MD001T). The library construction process included DNA fragmentation, end repair, adenylation (A-tailing), adapter ligation, and PCR amplification. All procedures were performed in strict accordance with the manufacturer’s protocols.

#### 2.4.3 Sequencing platform and parameters

For the sequencing phase of mNGS detection, the Illumina NextSeq 500 platform was uniformly employed using a 75-cycle single-end sequencing strategy. Through standardized sequencing protocols, each sample yielded approximately 10–20 million reads.

#### 2.4.4 Bioinformatics analysis pipeline

The bioinformatic analysis of mNGS data was performed according to the following workflow: First, reads were aligned to the human reference genome (hg19/GRCh37) using Bowtie2 to remove host-derived sequences. Subsequently, non-human reads were taxonomically classified with Kraken2 against the NCBI RefSeq database. These assignments were further validated by realigning reads to microbial RefSeq genomes using Bowtie2. In cases of discordant classification, BLAST (v2.9.0+) was employed for further verification. Following microbial identification, LOESS regression was applied to correct for GC bias. The corrected data were then analyzed with XHMM and Canoes for detection. Finally, a preliminary bioinformatic report was generated by bioinformaticians and interpreted by senior clinicians in the context of patient manifestations. A final clinical report was issued after excluding potential contaminants.

#### 2.4.5 Measures to ensure reproducibility

All experimental procedures were performed using commercial kits and automated systems, strictly in accordance with the manufacturer’s protocols. Bioinformatic analyses were conducted with publicly available software and databases (Kraken2, Bowtie2, BLAST, XHMM, Canoes), with version numbers explicitly specified.

### 2.5 Criteria for positive *cryptococcus* detection by mNGS

This study adopted a three-tiered criteria system—Core Metrics + Contamination Exclusion + Clinical Confirmation—to determine positive Cryptococcus neoformans mNGS results, as detailed below: a. Core Positive Criteria (ALL must be met):Read Count: The number of reads uniquely aligned to Cryptococcus neoformans must be ≥ 3.Coverage: The aligned reads must cover ≥ 1 unique genomic region (non-repetitive locus) of Cryptococcus neoformans, with a distribution pattern consistent with true infection. b. Contamination and Background Noise Exclusion: The read count or RPM value (reads per million) of the sample must be significantly higher than that of the same-batch negative control (e.g., sterile water), defined as: sample signal > mean of negative control + 3 standard deviations. The RPM value of the sample must exceed the 95th percentile of Cryptococcus neoformans in our laboratory’s background noise database. Stricter criteria are applied to non-sterile samples (e.g., sputum, bronchoalveolar lavage fluid (BALF)):Specific read count ≥ 5Coverage of ≥ 2 unique genomic regions. RPM value above the 99th percentile of the background database. c. Clinical Comprehensive Confirmation: The final positive result requires integrative interpretation by clinical physicians, incorporating mNGS data (read count, RPM value, etc.), patient clinical manifestations, laboratory findings (e.g., CrAg testing, fungal culture), and imaging results.

### 2.6 Validation of mNGS-detected pathogens

For all microorganisms detected in the mNGS report, we employ a tiered approach to verification and interpretation, with the level of scrutiny based on their clinical significance and their likelihood of being common colonizers or contaminants. For other potentially pathogenic microorganisms, the following criteria are applied: A. Clinical Relevance Assessment: All mNGS results are reviewed and discussed by multiple clinicians. Sequence data (read count and relative abundance) are evaluated alongside the patient’s clinical manifestations, imaging findings, and other laboratory tests—such as inflammatory markers and G/GM tests—to comprehensively classify each microorganism as a pathogen, colonizer, or environmental contaminant. B. Confirmatory Testing: For pathogens considered clinically relevant based on multidisciplinary discussion, retrospective verification using conventional methods is pursued whenever possible, including: Comparison with bacterial/fungal culture results from the same specimen type (e.g., BALF);Review of medical records for targeted PCR or RNA testing (e.g., for viruses such as CMV, EBV, influenza virus, or fastidious pathogens like Pneumocystis jirovecii);Review of whether serum IgM antibody testing was performed (e.g., for Mycoplasma pneumoniae or Legionella).

### 2.7 Statistical analysis

Statistical analyses were conducted using IBM SPSS Statistics, version 27.0. Continuous variables with a normal distribution are presented as mean ± standard deviation (SD), while non-normally distributed variables are reported as median with interquartile range [M (IQR)]. Given the paired study design, within-group comparisons were performed using the Wilcoxon signed-rank test. A two-tailed *p*-value of < 0.05 was considered statistically significant. No adjustments were made for multiple comparisons.

## 3 Results

### 3.1 Patient characteristics

#### 3.1.1 Demographic and clinical profiling of enrolled patients

Among the 31 patients diagnosed with pulmonary cryptococcosis in this study, 16 were male (51.61%) and 15 were female (48.39%), with ages ranging from 22 to 88 years (mean age: 59.52 years). Fourteen patients (45.16%) were aged over 60. Six patients (19.35%) were admitted to the intensive care unit (ICU), with 3 (9.68%) requiring mechanical ventilation. Prior to hospitalization, six patients (19.35%) had received oral immunosuppressive therapy (glucocorticoids, tacrolimus), of these, 2 (6.45%) were admitted to the ICU (**Table 1**). Regarding hospitalization duration, 14 patients (45.16%) had brief hospital stays of 10 days or fewer, while 1 patient (3.23%) experienced a prolonged stay exceeding 30 days (**Table 1**). Four patients (12.90%) were asymptomatic. Among symptomatic individuals, the most commonly reported clinical manifestation was cough (61.30%), followed by fever (51.61%), chest tightness (41.94%), chest pain (12.90%), hemoptysis/blood-tinged sputum (9.68%), and headache (3.23%) (**Table 1**). Underlying medical conditions were present in 74.19% of the study population, including chronic lung diseases (such as idiopathic pulmonary fibrosis), postoperative conditions (such as lung tumors, endocrine tumors, and gynecological tumors), as well as other comorbidities (such as hypertension, diabetes, and connective tissue diseases) (**Table 1**). All 31 cases were diagnosed with pulmonary cryptococcosis. No cases of cryptococcal meningitis or extrapulmonary involvement were observed (**Table 1**).

**Table 1:** The demographic and clinical profiling of the enrolled patients.

Characteristics	Total (n=31)
Age, mean ± sd	59.52 ± 15.24
Gender, no. (%)	
Male	16 (51.61
Female	15 (48.39)
Smoke, no. (%)	8 (25.81)
Immunosuppressants, no. (%)	6 (19.35)
ICU, no. (%)	6 (19.35)
Ventilator, no. (%)	3 (9.68)
Hospital days, no. (%)	
<10 days	14 (45.16)
10-30days	16 (51.61)
>30days	1 (3.23)
Main symptoms, no. (%)	
Cough	19 (61.30)
Fever	16 (51.61)
Chest tightness	13 (41.94)
Chest pain	4 (12.90)
Hemoptysis/Blood-streaked sputum	3 (9.68)
Headache	1 (3.23)
Asymptomatic	4 (12.90)
Underlying diseases, no. (%)	
Hypertension	13 (41.94)
Diabetes	10 (32.26)
Chronic lung disease	1 (3.23)
Connective tissue disease	2 (6.45)
Lung cancer	3 (9.68)
Other malignant tumors	6 (19.35)
Nephrotic Syndrome	2 (6.45)
Syphilis	1 (3.23)
Post-renal transplant	1 (3.23)
Without underlying medical conditions	8 (25.81)
Cryptococcosis infection sites, no. (%)	
Pulmonary	31 (100.00)
CNS	0 (0.00)

#### 3.1.2 Chest CT imaging characteristics

The chest CT manifestations of pulmonary cryptococcosis are highly diverse and lack specific imaging features. The typical imaging findings can be categorized into the following four main types: (1) Nodular Type (Most Common): Characterized by single or multiple nodules, predominantly located subpleurally and in the lower lung fields. These nodules are typically well-defined and often measure less than 2 cm in diameter (Huang et al., 2024). (2) Pneumonic Type (Large Consolidation Type): More frequently observed in immunocompromised patients or those with rapid disease progression. Lesions commonly appear as consolidations with a broad base attaching to the pleura, often in the lower lobes. Associated pleural thickening may be present, and some lesions may exhibit air bronchograms.(3) Cavitary Type (Relatively Uncommon): Primarily found in the lower lobes. Cavities usually develop from pre-existing nodules or areas of consolidation. The cavity walls are often ill-defined but smooth on the inner surface, and typically lack an air-fluid level.(4) Mixed Type: Defined by the simultaneous presence of two or more of the above lesion types. The combination of consolidation with nodules is frequently observed. Most lesions remain distributed in subpleural regions (Chang et al., 2006; Xiong et al., 2022; Huang et al., 2024).

Chest computed tomography (CT) was used to evaluate pulmonary involvement among the study cohort. Of the 31 patients included, 27 had accessible in-hospital CT imaging data, while 4 patients underwent CT scans at external facilities. Characteristic imaging features associated with pulmonary cryptococcosis include the tree-in-bud sign, air bronchogram sign, broad-based sign, spicule sign, cavitation sign, linear opacities along lesion margins, and pleural traction sign. **Supplementary Table 1** summarizes imaging features in 31 patients (presented as **Supplementary Material** due to extensive data; refer to **Supplementary Table 1** in Supplementary Material for details). CT scan analysis revealed that most patients exhibited multiple patchy, fluffy, or nodular lesions located near the pleura, while a minority presented with mass-like lesions (**Supplementary Table 1**). Among the 27 patients with in-hospital scans, 25 (92.59%) presented with multiple lesions, whereas only 2 (7.41%) had solitary lesions. Additionally, 7 patients (25.93%) showed cavitation and 12 patients (44.44%) had accompanying hilar/mediastinal lymphadenopathy. Four patients (14.81%) presented with pulmonary atelectasis, including 1 case each of right middle lobe syndrome and lung cancer. Notably, 11 patients (40.74%) presented with pleural effusion, and 11 patients (40.74%) exhibited air bronchogram sign. Analysis of lesion distribution (N = 27) revealed a predominance of multilobar involvement, with bilateral multilobar lesions being the most frequent (55.56%, 15/27), accounting for 93.75% of all multilobar cases. Unilateral lesions comprised 33.33% (9/27), predominantly right-sided (18.52% vs 14.81% on the left). Lower lobe lesions (29.63%) were primarily unilateral left-sided (4/8 cases), while upper lobe involvement was rare (11.11%). Critically, no middle lobe involvement was observed in any case. This distribution pattern suggests that bilateral multilobar involvement may be a hallmark feature in this cohort, potentially linked to disease progression or underlying pathological mechanisms.

### 3.2 Results for mNGS

#### 3.2.1 Comparison of turnaround time and result time between mNGS and fungal culture in BALF

**Figure 2:**
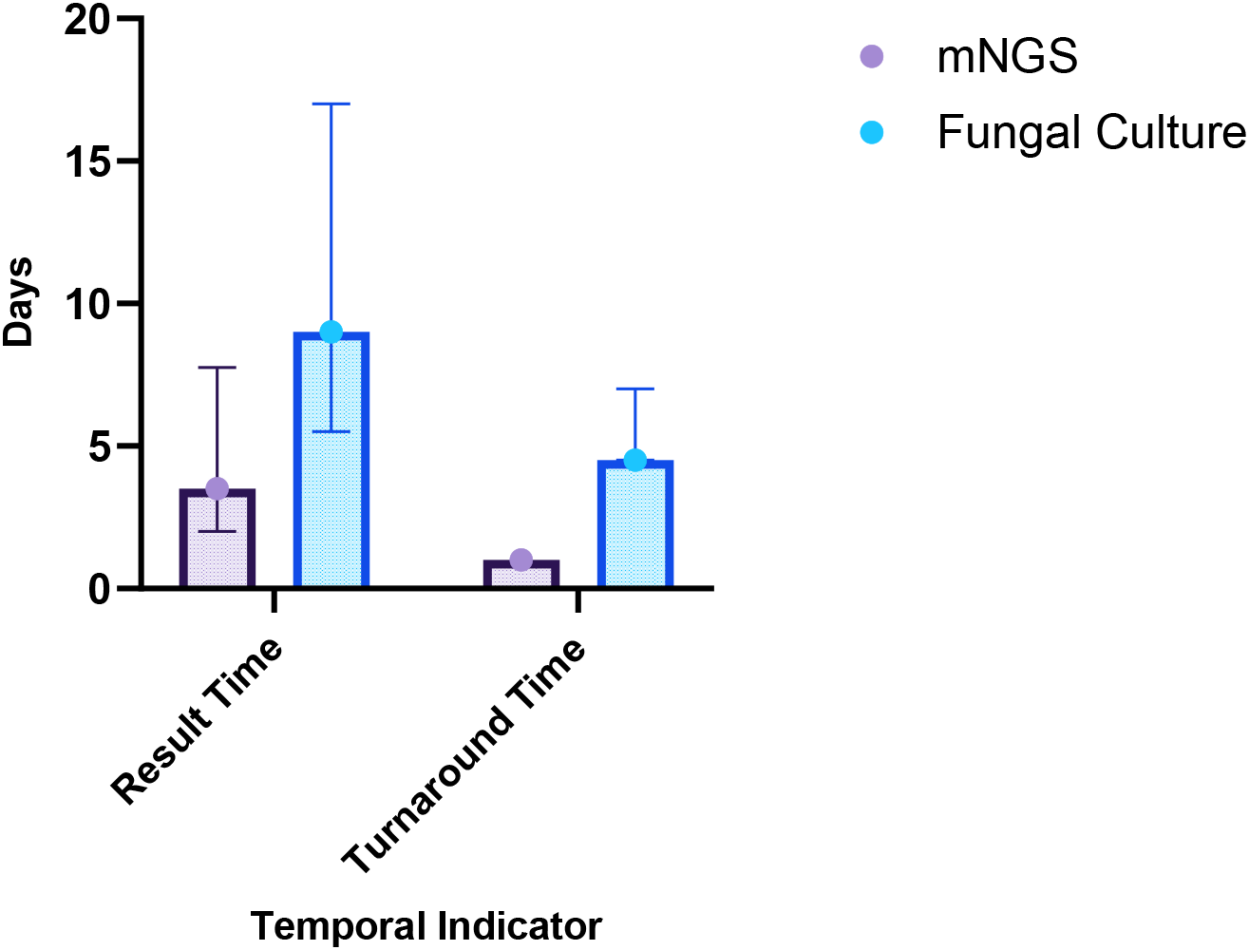
Comparison of result time and turnaround time between mNGS and fungal culture.

Among the 31 patients, 12 underwent both mNGS and fungal culture of BALF simultaneously, resulting in 12 paired samples for comparative analysis. The Wilcoxon signed-rank test was used to compare result time and turnaround time between the two methods, as the data were not normally distributed. Accordingly, the results are reported as median and interquartile range [M (IQR)]. As shown in **Table 2**, the median result time for mNGS was 3.50 days (IQR: 2.00–7.75), significantly shorter than that for fungal culture, which was 9.00 days (IQR: 5.50–17.00) (*p* = 0.002). Similarly, the median turnaround time for mNGS was 1.00 day (IQR: 1.00–1.00), notably less than that or fungal culture (4.50 days, IQR: 4.50–7.00) (*p* = 0.002) (**Figure 2**). These findings indicate that mNGS offers a significantly faster diagnostic timeline compared to conventional fungal culture, enhancing the timeliness of clinical decision-making.

**Table 2:** The comparison of turnaround time and result time between mNGS and fungal culture in BALF.

Detection Method	Result time [M (IQR)], days	Turnaround time [M (IQR)], days
mNGS	3.50 (2.00-7.75)	1.00 (1.00-1.00)
Fungal Culture	9.00 (5.50-17.00)	4.50 (4.50-7.00)
P value	0.002	0.002

#### 3.2.2 Distribution of infection types in patients with cryptococcal infection detected by mNGS

**Figure 3:**
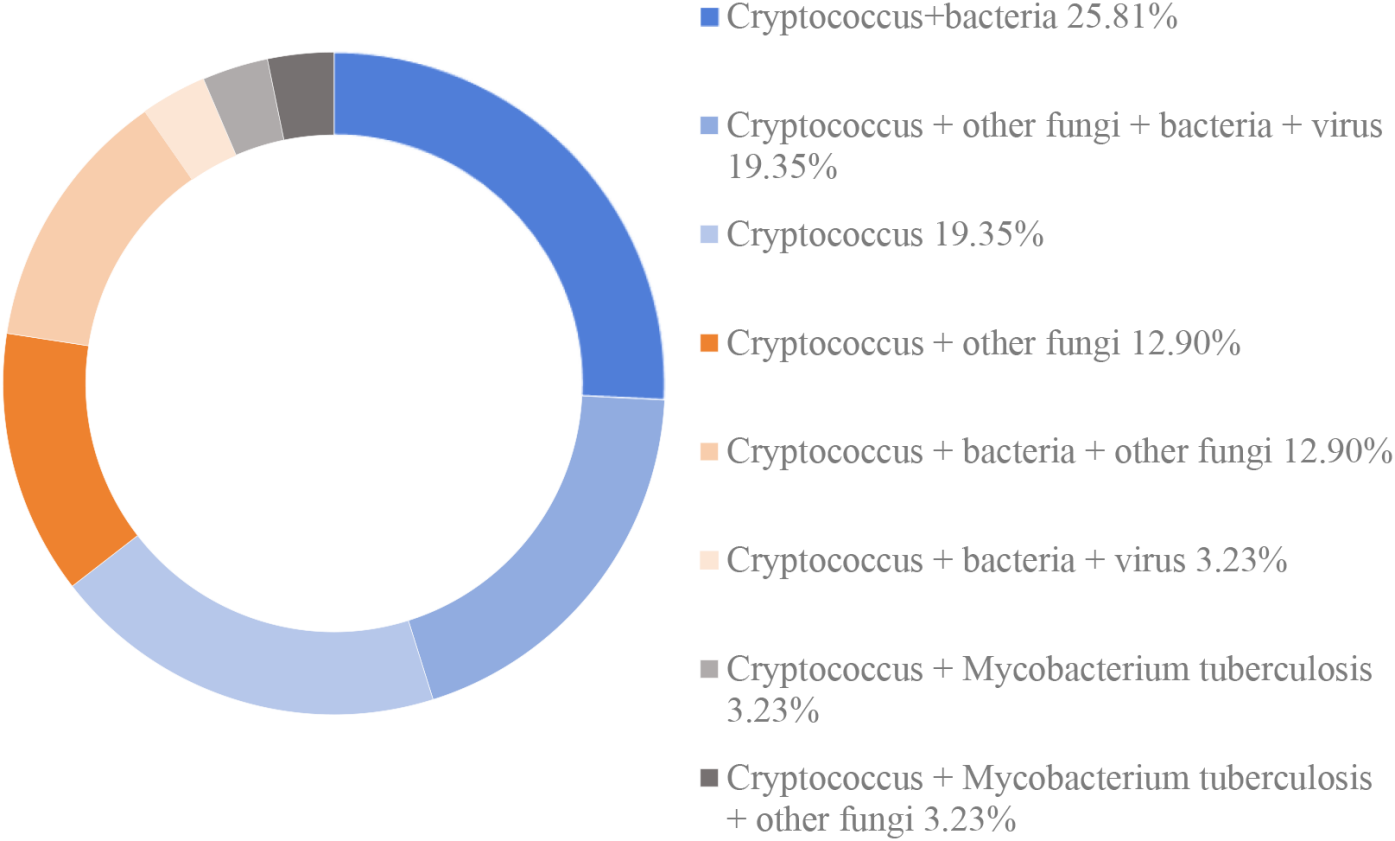
The distribution of infection types in patients with cryptococoal infection detected by mNGS.

Among the mNGS-detected cases, 6 patients (19.35%) had mono-infection with *Cryptococcus*, while the remaining 25 patients (80.65%) exhibited co-infections, defined as the simultaneous presence of *Cryptococcus* and one or more additional pathogens, including bacteria, fungi, or viruses. The most prevalent co-infection pattern was *Cryptococcus* + bacteria (25.81%, 8/31). This was followed by *Cryptococcus* + other fungi + bacteria + viruses (19.35%, 6/31), *Cryptococcus* + bacteria + other fungi (12.90%, 4/31), *Cryptococcus* + other fungi (12.90%, 4/31), *Cryptococcus* + bacteria + viruses (3.23%, 1/31), *Cryptococcus* + *Mycobacterium tuberculosis* (3.23%, 1/31), and *Cryptococcus* + *Mycobacterium tuberculosis* + other fungi (3.23%, 1/31) (**Figure 3**). Notably, all cryptococcal infections identified through mNGS were attributed exclusively to *Cryptococcus neoformans*.

#### 3.2.3 Comparison of microorganism detection between BALF-mNGS and traditional detection methods

In this study, BALF samples were used to systematically evaluate the diagnostic efficacy of mNGS and traditional detection methods—including fungal culture, immunofluorescence staining, and Gomori methenamine silver (GMS) staining—for pulmonary cryptococcosis. In the detection of 26 samples, mNGS technology demonstrated exceptional detection capabilities. The detection rate for *Cryptococcus* reached 100% (26/26), for other fungi it was 57.70% (15/26). In contrast, traditional detection methods showed significantly lower detection rates. For fungal culture (12 samples), the *Cryptococcus* detection rate was only 8.33% (1/12), the other fungi detection rate was 41.67% (5/12). Using immunofluorescence staining (23 samples), the *Cryptococcus* detection rate was 0% (0/23), the other fungi detection rate was 4.35% (1/23). With Gomori methenamine silver (GMS) staining (15 samples), the detection rates for *Cryptococcus*, other fungi were all 0% (0/15). These data conclusively indicate that mNGS technology significantly outperforms traditional methods in sensitivity for detecting *Cryptococcus* and other fungi. (**Table 3**).

**Table 3:** The comparison of microorganism detection between BALF-mNGS and traditional detection methods in patients with pulmonary cryptococcosis.

Detection Method	The detection rate of *Cryptococcus* (n/N)	The detection rate of other fungi (n/N)	The detection rate of fungi (n/N)
mNGS	26/26 (100%)	15/26 (57.70%)	26/26 (100%)
Fungal culture	1/12 (8.33%)	5/12 (41.67%)	6/12 (50%)
Immunofluorescence staining	0/23 (0%)	1/23 (4.35%)	1/23 (4.35%)
Gomori methenamine silver (GMS) staining	0/15 (0%)	0/15 (0%)	0/15 (0%)

The detection rate of *Cryptococcus* (n/N): The percentage of samples in which *Cryptococcus* was detected. The detection rate of other fungi (n/N): The percentage of samples in which any non-Cryptococcus fungal species (e.g., potentially co-infecting pathogens such as *Aspergillus* or *Candida*) was detected. The detection rate of fungi (n/N): The percentage of samples in which any fungal species (including both *Cryptococcus* and other fungi) was identified. This value is derived from the sum of the detection rate of *Cryptococcus* and the detection rate of other fungi.

### 3.3 Treatment and prognosis

Antimicrobial treatment regimens administered before and after mNGS testing were reviewed for all 31 patients. Prior to mNGS analysis, antimicrobial therapy provided effective coverage for the identified pathogens in only 1 patient (**Table 4**). Following mNGS-guided therapeutic adjustments, 26 patients (83.87%) received targeted antimicrobial therapy aligned with the identified pathogens. The remaining 5 patients did not receive additional antitubercular or antiviral therapy due to either the detection of Mycobacterium tuberculosis (requiring specialized treatment beyond the study’s scope) or the detection of viral sequences below established clinical reporting thresholds.

**Table 4:** The changes in the usage of antibacterial drugs for all patients before and after mNGS.

Treatment Phase	Cases using Fluconazole/Amphotericin B no. (%)	Cases covering pathogens no. (%)	Cases using restricted-class antibiotics no. (%)
Before mNGS	0	1 (3.23%)	0
After mNGS	27 (87.10%)	26 (83.87%)	13 (41.94%)

Among the 31 patients, 27 (81.10%) received fluconazole or amphotericin B lipid complex, indicating the implementation of mNGS-guided tailored therapeutic interventions. The remaining 4 patients received voriconazole as part of their post-mNGS regimen. Notably, restricted-spectrum antimicrobial agents were introduced in 13 patients (41.94%) following mNGS analysis (**Table 4**).

**Figure 4:**
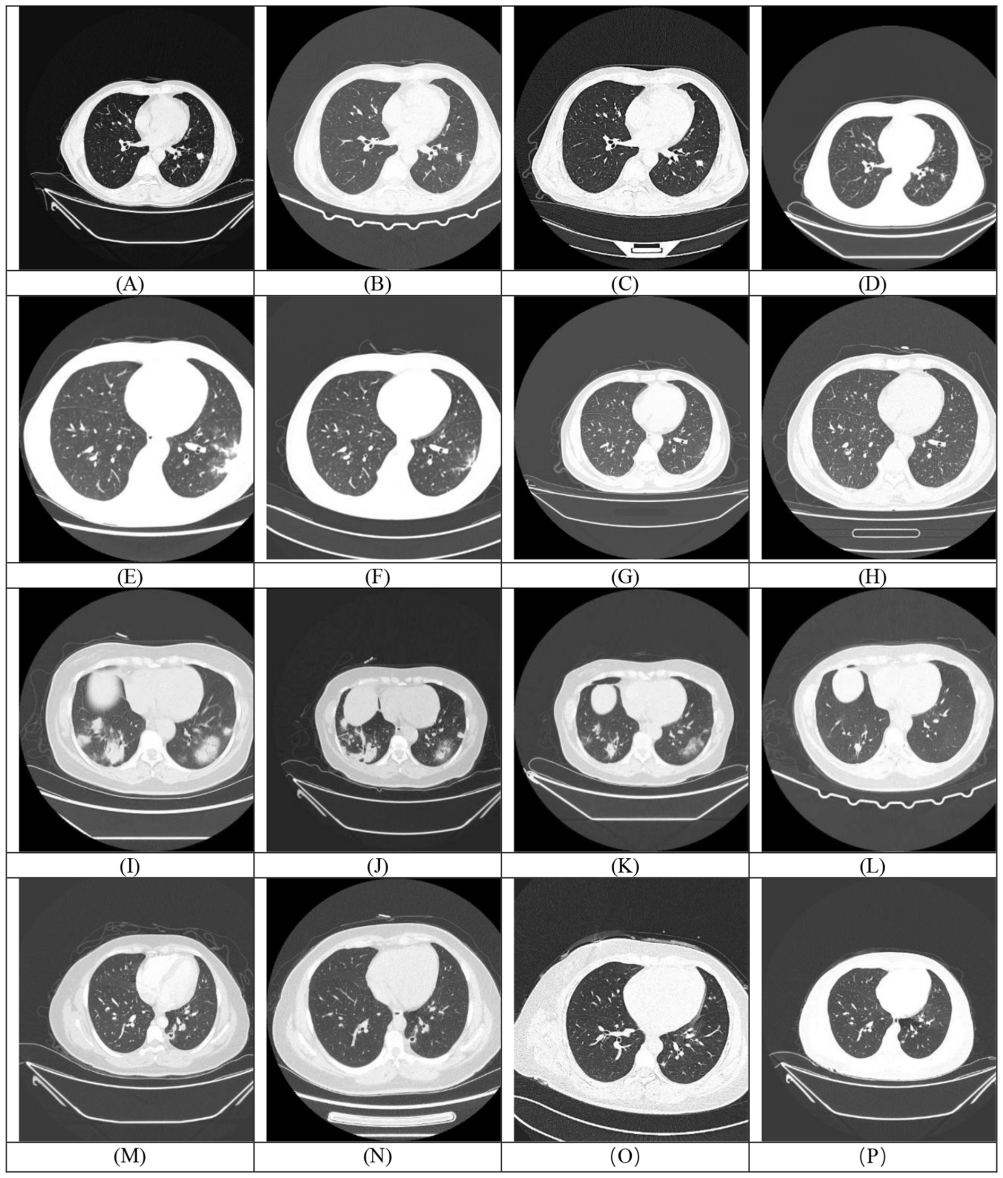
Each row (Rows 1-4) corresponds to a single patient. The first image in each row **(A, E, I, M)** represents the baseline (pre-treatment) scan, while the subsequent images **(B-D, F-H, J-L, N-P)** are post-treatment follow-up scans demonstrating temporal changes in the lesions.

Among the 31 enrolled patients, 4 (12.50%) died during hospitalization. Of the 27 survivors, follow-up revealed 1 case (3.70%) of disease recurrence, one death (3.70%) from brain metastasis secondary to a lung tumor, and 1 patient (3.70%) lost to follow-up. Most patients undergoing repeat chest CT scans within one to three months of initial evaluation. Among the patients with follow-up CT imaging data (n=14), the comparison of CT manifestations before and after treatment in some cases is shown in **Figure 4**. Among these 14 patients, 3 cases (21.43%) achieved complete lesion resolution, 7 (50.00%) exhibited sustained improvement with reduced lesion size, 3 (21.43%) showed partial radiographic improvement, and only 1 patient (7.14%, 1/14) experienced disease progression.

## 4 Discussion

In this retrospective study, our primary objective was to evaluate the clinical utility of mNGS in guiding targeted therapy following the detection of *Cryptococcus*. We enrolled 31 patients with lower respiratory tract infections (LRTIs) in whom *Cryptococcus* was identified via mNGS. A comprehensive analysis of clinical characteristics, imaging features, underlying diseases, treatment regimens, prognoses, and laboratory tests results (including conventional microbiological examinations results) were conducted. Consistent with previous studies (Xie et al., 2024), compared to fungal culture, mNGS demonstrated a significantly shorter turnaround time (TAT), indicating its potential to reduce the time from hospital admission to clinical decision-making and mitigate the risk of treatment delays. Furthermore, mNGS exhibited a higher positive predictive value (PPV) than conventional microbiological testing, aligning with earlier findings (Fang et al., 2020). Compared to conventional microbiological methods, another key advantage of mNGS is its ability to simultaneously detect multiple microorganisms, making it particularly effective for diagnosing complex or atypical infections (Wang et al., 2019). This broad-spectrum detection allows more timely and informed clinical decision-making.

Pulmonary cryptococcosis is often challenging to diagnose due to its nonspecific clinical and radiological features and the low sensitivity of culture-based tests. Misdiagnoses as lung cancer, pulmonary tuberculosis, or other pulmonary infections are common, especially when symptoms are mild or overlap with other conditions (Zavala and Baddley, 2020). In our cohort, clinical presentations ranged from asymptomatic cases (12.90%) to severe respiratory compromise, including cough, fever, chest tightness, chest pain, hemoptysis/blood-tinged sputum, and headache. In addition, 74.19% of patients had at least one underlying condition, such as comorbidities (diabetes, hypertension, connective tissue diseases), interstitial lung disease, or postoperative tumor state. Given this diagnostic uncertainty, mNGS has been increasingly used in the diagnosis and treatment of lower respiratory tract infectious diseases (Huang et al., 2024). mNGS, as a non-biased detection method, can simultaneously sequence a large amount of DNA and facilitate the detection of a broad range of microorganisms within a single assay (Gu et al., 2019). This is particularly beneficial for immunocompromised or polymorbid patients, who are at higher risk for co-infections. In our study, only 6 patients had solitary Cryptococcal infection, while the remaining 25 had concomitant bacterial, viral, or fungal infections. Traditional pathogen detection methods often fail to identify such mixed infections due to their limited sensitivity and narrow pathogen scope.

Previous studies have indicated that the positive detection rate of *Cryptococcus* cultures is susceptible to interference from multiple factors. On one hand, the standardization of specimen collection and sample quality significantly impact the results (Loyse et al., 2013, McCarthy and Walsh, 2017, World Health Organization, 2017). On the other hand, prior antifungal therapy in patients may suppress fungal viability, markedly reducing the likelihood of obtaining positive culture results (Perfect et al., 2010). In this study, fungal cultures of BALF were performed in 12 patients, with *Cryptococcus neoformans* isolated in only one case. Although BALF-mNGS is an invasive procedure, it offers a less harmful and more accessible alternative to tissue biopsy (Du Rand et al., 2013), especially when lesions are inaccessible by a tissue biopsy, when facilities lack biopsy capabilities, or when rapid etiological diagnosis is urgently needed.

Among the 31 patients, 27 (87.10%) initiated targeted treatment for cryptococcosis with fluconazole or liposomal amphotericin B on the same day the mNGS results became available, while 4 others received voriconazole. Follow-up data revealed a favorable response in most cases, with only one patient experiencing relapse, and none of the patients progressed to cryptococcal meningitis. These outcomes highlight the clinical value of mNGS in enabling early, pathogen-specific therapy. Although prospective evidences are still needed to confirm the impact of mNGS use on long-term outcomes, our findings suggest that mNGS can reduce diagnostic delays, enhance treatment precision, and potentially improve prognosis.

This study has several limitations, including its single-center retrospective design, limited sample size (n=31), and the fact that not all patients underwent CrAg testing, making it difficult to use CrAg as a gold standard for comparison or to calculate the specificity and predictive values of mNGS. Furthermore, mNGS cannot distinguish between contamination, colonization, and true infection, emphasizing the importance of interpreting results in the context of the patients’ clinical presentation (Salter et al., 2014). Nevertheless, this study demonstrates that BALF mNGS exhibits high sensitivity in patients with suspected pulmonary cryptococcosis, significantly outperforming traditional fungal culture and pathological examinations. For patients from whom pathological specimens cannot be obtained or whose culture results are negative, mNGS may serve as an efficient supplementary diagnostic tool and even a potential first-line option. Future prospective studies involving broader patient cohorts are needed to systematically compare the performance of mNGS and CrAg and comprehensively evaluate the diagnostic accuracy of mNGS.

## 5 Conclusion

In our study, we evaluated the clinical characteristics and infection profiles of 31 patients with Cryptococcus detected by mNGS. Compared to fungal culture, mNGS demonstrated a significantly shorter TAT, facilitating earlier diagnosis. More importantly, mNGS enabled the detection of Cryptococcus alongside concurrent polymicrobial co-infections, supporting its value in diagnosing complex infections that may be missed by conventional tests. Early initiation of antifungal therapy based on mNGS results was associated with significantly improved clinical outcomes in these patients, emphasizing the potential of mNGS to improve the precision and effectiveness of therapy in pulmonary cryptococcosis.

## Data Availability

The original contributions presented in the study are included in the article/**Supplementary Material**. Further inquiries can be directed to the corresponding authors.

## Supplementary material

Supplementary Table 1 The chest computed tomography imaging characteristics of 31 patients. (Due to the extensive data volume, detailed imaging findings are provided as supplementary material; refer to **Supplementary Table 2** for comprehensive results).
